# Minimum ADC outperforms PSA and PSA density for predicting ISUP grade group in prostate cancer: a retrospective study of 192 patients undergoing radical prostatectomy

**DOI:** 10.3389/fonc.2026.1905995

**Published:** 2026-09-21

**Authors:** Katarzyna Skrobisz, Weronika Bernard, Rafał Bromirski, Karolina Nowak, Anna Glińska, Wojciech Połom, Joanna Pieńkowska

**Affiliations:** 1 Department of Radiology, Medical University of Gdańsk, Gdańsk, Poland; 2 2nd Department of Radiology, Medical University of Gdańsk, Gdańsk, Poland; 3 Department of Urology, Medical University of Gdańsk, Gdańsk, Poland

**Keywords:** Apparent diffusion coefficient, Diffusion-weighted imaging, Gleason Score, ISUP grade group, Multiparametric MRI, prostate cancer, PSA density, risk stratification

## Abstract

**Background:**

Apparent diffusion coefficient (ADC) derived from diffusion-weighted imaging (DWI) has been proposed as a quantitative biomarker of prostate cancer aggressiveness, and an inverse relationship between ADC and Gleason score has been reported across numerous single-marker studies and meta-analyses. However, most prior work has compared ADC with serum PSA alone, without accounting for PSA density (PSAD) or potential confounders such as age and prostate volume, and without formally testing — using multivariable regression and receiver operating characteristic (ROC) analysis — whether ADC provides incremental diagnostic value beyond PSA-based parameters. Whether the ADC–grade relationship persists after such adjustment, and whether combining ADC with PSA and PSAD meaningfully improves discrimination, remains inconsistently characterized.

**Objective:**

To evaluate whether minimum apparent diffusion coefficient (ADC) values are associated with pathological International Society of Urological Pathology (ISUP) Grade Group, and to compare the diagnostic performance of ADC with that of serum PSA and PSA density (PSAD), in patients with histologically confirmed prostate cancer undergoing radical prostatectomy.

**Methods:**

A retrospective study of 192 patients undergoing multiparametric MRI (mpMRI), biopsy, and radical prostatectomy was performed. Minimum ADC values were correlated with the final pathological ISUP Grade Group, serum PSA, PSA density, age, and prostate volume.

**Results:**

Minimum ADC showed a significant inverse correlation with ISUP Grade Group (Spearman’s ρ = −0.355; 95% CI, −0.473 to −0.218; p < 0.001), whereas neither PSA (ρ = 0.012; p = 0.864) nor PSAD (ρ = 0.040; p = 0.578) showed a significant monotonic relationship. On multivariable ordinal logistic regression, minimum ADC remained independently associated with higher ISUP Grade Group after adjustment for age, PSA or PSAD, and prostate volume (OR = 0.995 per 1 × 10^−6^ mm²/s increase; p < 0.001 in both models), while age, PSA, PSAD, and prostate volume were not independently significant. Minimum ADC discriminated ISUP Grade Group 1 from ≥2 with an AUC of 0.959 (95% CI, 0.923–0.988), substantially outperforming PSA (AUC = 0.586) and PSAD (AUC = 0.562). Combined models incorporating ADC with PSA and/or PSAD did not significantly improve discrimination over ADC alone (DeLong’s test, all p ≥ 0.10).

**Conclusion:**

In this radical prostatectomy cohort, minimum ADC was inversely and independently associated with pathological ISUP Grade Group, whereas serum PSA and PSAD were not. ADC substantially outperformed PSA-based markers for discriminating low- from higher-grade disease, although combining ADC with PSA-based parameters provided no significant incremental benefit. These findings support ADC as a promising quantitative imaging biomarker of tumor grade; however, given the retrospective, single-center, surgically selected design and the small number of ISUP Grade Group 1 cases, prospective multicenter validation is required before ADC-based thresholds can be recommended for active surveillance or biopsy decision-making.

## Introduction

Prostate cancer (PCa) remains one of the leading causes of male cancer morbidity and mortality worldwide (1, 2). The rising incidence in many populations reflects increasing life expectancy, lifestyle factors, and broader use of prostate-specific antigen (PSA) screening. Although mortality is declining in many regions, total case numbers continue to expand as early detection increases. Prostate cancer has thus evolved from a rare to a highly scrutinized disease, often diagnosed at asymptomatic stages.

PSA testing, introduced in the late 1980s, revolutionized early detection of prostate cancer (3). PSA, a serine protease secreted by prostatic epithelial cells, serves as a surrogate measure of both normal and malignant glandular activity. However, its clinical interpretation is limited by low specificity and substantial inter-individual variability (4, 5). Elevated concentrations may occur in benign prostatic hyperplasia (BPH) or prostatitis, whereas high-grade tumors may exhibit low PSA secretion due to loss of glandular differentiation and reduced luminal secretion (6). This paradox of “low PSA, high-grade disease” has reduced reliance on PSA as a sole parameter in risk assessment.

To improve specificity, PSA density (PSAD)—calculated as PSA divided by prostate volume—was introduced. PSAD adjusts PSA according to gland size and increases detection specificity for clinically significant disease, particularly in men with intermediate PSA levels. A threshold of approximately 0.15 ng/mL/cm³ identifies higher malignancy risk (7, 8). Despite advances in MRI, PSAD remains a simple yet effective adjunct for refining biopsy decisions and differentiating indolent from significant disease.

Histological grading through the Gleason system, established in 1966 and later standardized by the ISUP Grade Group classification, remains the most powerful prognostic marker in PCa (9). The sum of the two predominant glandular patterns (scored 1–5) defines the Gleason grade, closely linked to genomic instability and biological behavior (10). Low-grade (≤6) lesions maintain glandular architecture and often follow an indolent course, while high-grade (≥8) lesions show disorganized, fused glands and increased proliferation, correlating with poor clinical outcomes.

Multiparametric magnetic resonance imaging (mpMRI) has re-defined the diagnostic pathway of prostate cancer. In particular, diffusion-weighted imaging (DWI) and the derived apparent diffusion coefficient (ADC) quantify the movement of water molecules within tissue, providing insight into microstructural arrangement (11, 12). ADC inversely correlates with cell density and loss of glandular lumina, both hallmarks of dedifferentiation. Lower ADC values correspond to higher Gleason grades and greater tumor aggressiveness, as shown in multiple 3 T MRI studies and meta-analyses (13–15).

Recent research also indicates that markedly reduced ADC in nominally low-grade (3 + 3) lesions may reveal undergrading on biopsy, helping identify patients who require confirmatory investigation rather than immediate active surveillance (7, 16). By combining ADC with PSA and PSAD, clinicians may obtain a composite view that captures biochemical activity, morphological transformation, and architectural disruption—bridging the gap between imaging and histopathology.

This study therefore aimed to evaluate the relationship between minimum ADC, pathological ISUP Grade Group, PSA, and PSAD in patients with histologically proven prostate cancer undergoing radical prostatectomy, assessing whether ADC more accurately reflects biological tumor aggressiveness than serum-based markers alone, and whether it provides incremental diagnostic value when combined with PSA-based parameters.

## Materials and methods

### Study design and cohort

We conducted a single-center retrospective study of patients who underwent multiparametric MRI (mpMRI) of the prostate between 2023 and 2025. All imaging was performed prior to biopsy and radical prostatectomy, allowing direct clinicopathological correlation.

The study cohort included 192 men with histologically confirmed prostate cancer who underwent radical prostatectomy. Final pathological grading was reported according to the 2014 ISUP consensus Grade Group system, which consolidates the Gleason score into five prognostic groups: Grade Group 1 (Gleason 3 + 3), Grade Group 2 (Gleason 3 + 4), Grade Group 3 (Gleason 4 + 3), Grade Group 4 (Gleason 4 + 4, 3 + 5, or 5 + 3), and Grade Group 5 (Gleason 4 + 5 or 5 + 4) (10). Representative cases illustrating the spectrum of ADC values across tumor grades are shown in **Figures 1**-**4**, corresponding to final pathological Gleason scores of 4 + 3 (**Figure 1**), 3 + 4 (**Figure 2**), 4 + 3 (**Figure 3**), and 3 + 3 (**Figure 4**). The distribution of ISUP Grade Groups within the cohort is shown in **Table 1**. Baseline demographic and clinical characteristics, including age, PSA, and PSA density stratified by ISUP Grade Group, are summarized in **Table 2** and described in detail in the Results section.

**Figure 1:**
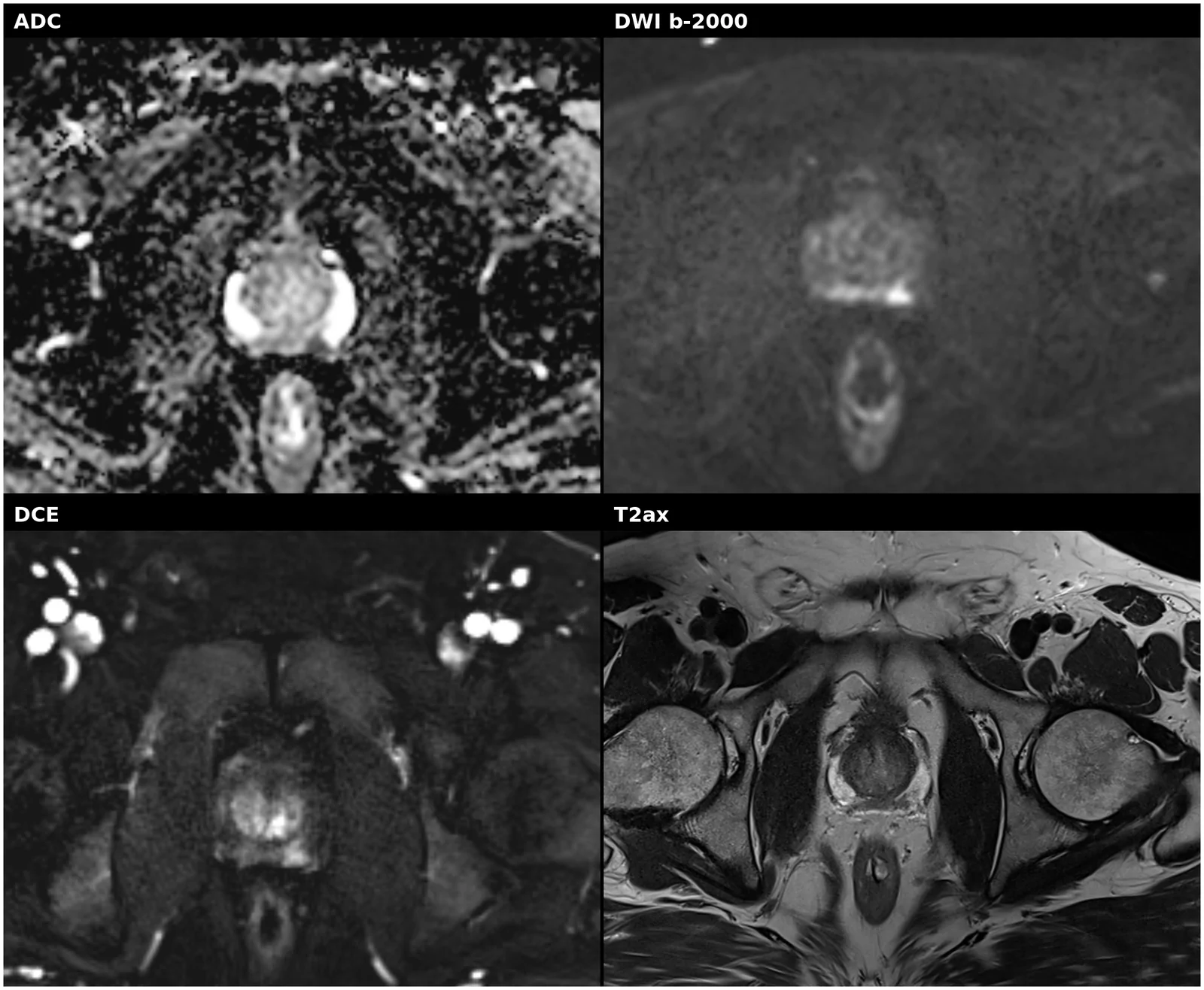
Multiparametric MRI of the prostate demonstrating a focal lesion in the left peripheral zone at the mid-gland level. The patient was 56 years old with a serum PSA level of 4.1 ng/mL and a PSA density (PSAD) of 0.10 ng/mL/cm³. Fusion-guided biopsy revealed prostate adenocarcinoma with a Gleason score of 3 + 4 (Grade Group 2). Final histopathological examination following robot-assisted radical prostatectomy (RARP) demonstrated a Gleason score of 4 + 3 (Grade Group 3). The lesion showed restricted diffusion with a mean ADC value of 629 × 10^−6^ mm²/s (range: 520–720 × 10^−6^ mm²/s). MRI sequences shown include ADC map, high b-value DWI (b = 2000 s/mm²), dynamic contrast-enhanced imaging (DCE), and axial T2-weighted imaging (T2ax).

**Figure 2:**
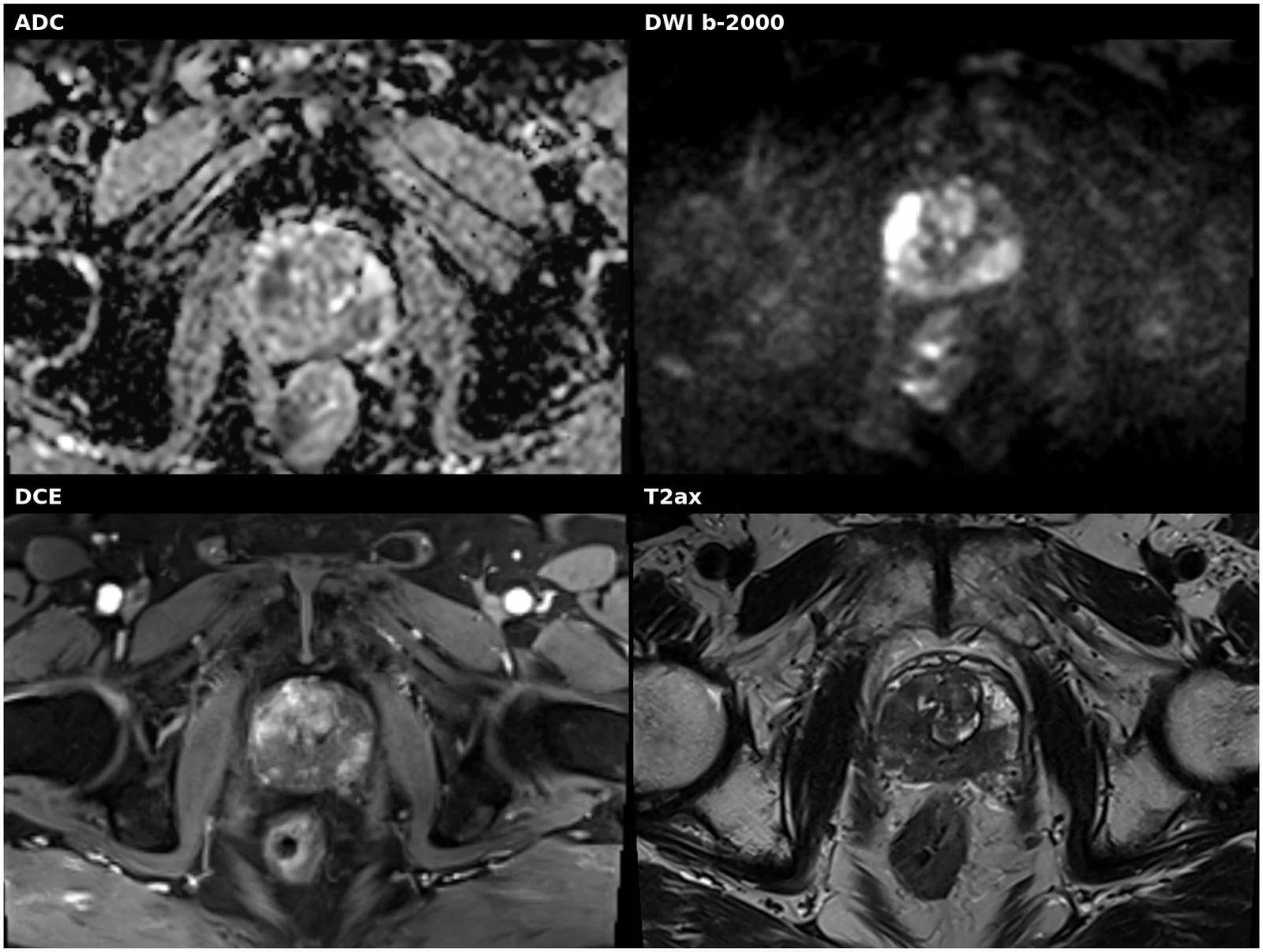
Multiparametric prostate MRI demonstrating a dominant focal lesion in the peripheral zone of the right lobe at the mid-gland level. The patient was 67 years old with a serum PSA level of 9.18 ng/mL and a PSAD of 0.417 ng/mL/cm³. Fusion-guided biopsy revealed prostate adenocarcinoma with a Gleason score of 4 + 4 (Grade Group 4). Final histopathological examination following RARP demonstrated a Gleason score of 3 + 4 (Grade Group 2). The lesion showed restricted diffusion with a mean ADC value of 690 × 10^−6^ mm²/s (range: 530–814 × 10^−6^ mm²/s). MRI sequences: ADC map, DWI (b = 2000 s/mm²), DCE, T2ax.

**Figure 3:**
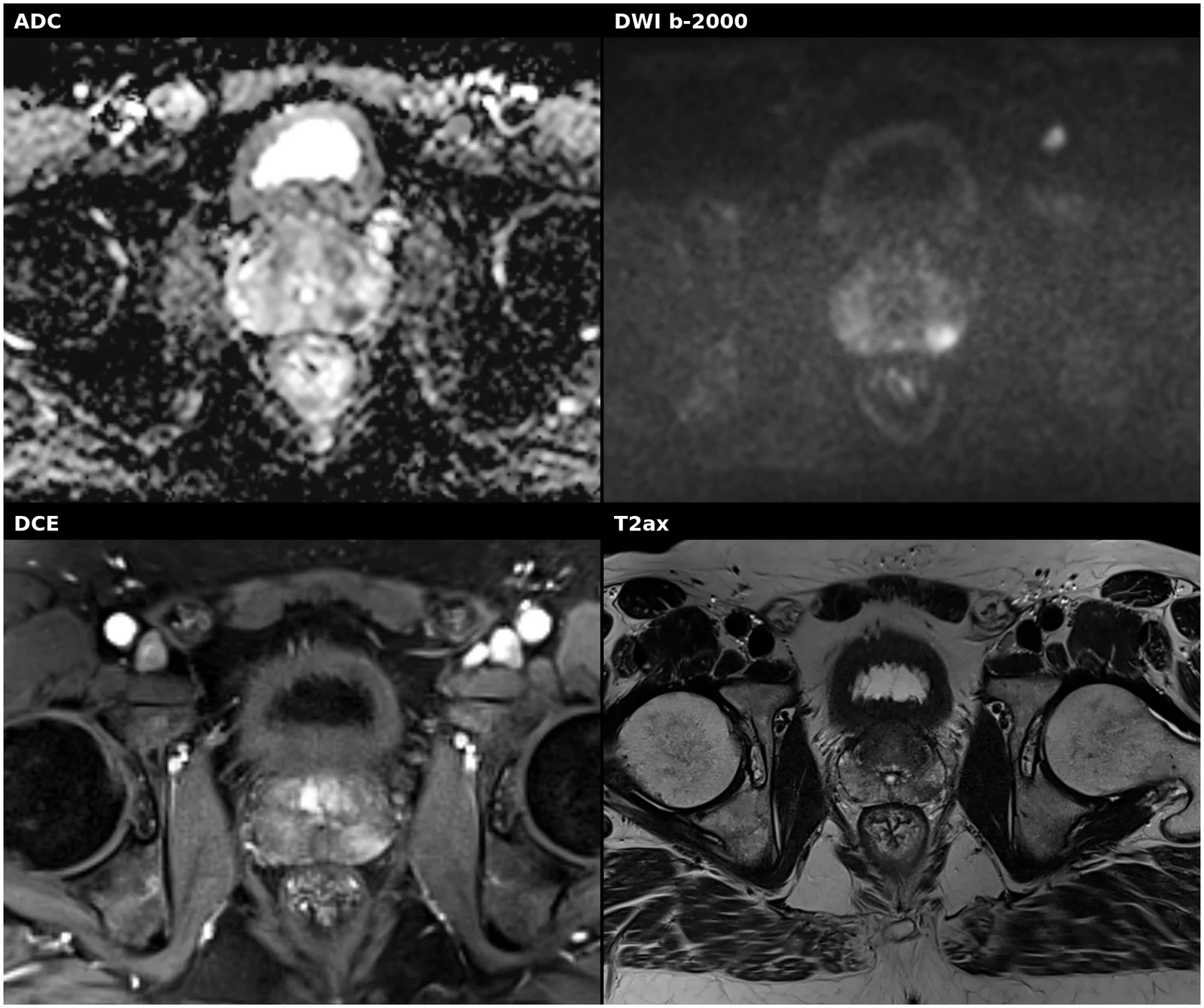
Multiparametric prostate MRI demonstrating a focal lesion in the peripheral zone of the left lobe at the mid-gland level, extending towards the base. The patient was 57 years old with a serum PSA level of 5.9 ng/mL and a PSAD of 0.125 ng/mL/cm³. Fusion-guided biopsy revealed prostate adenocarcinoma with a Gleason score of 3 + 3 (Grade Group 1). Final histopathological examination following RARP demonstrated a Gleason score of 4 + 3 (Grade Group 3). The lesion showed marked restricted diffusion with a mean ADC value of 513 × 10^−6^ mm²/s (range: 417–606 × 10^−6^ mm²/s). MRI sequences: ADC map, DWI (b = 2000 s/mm²), DCE, T2ax.

**Figure 4:**
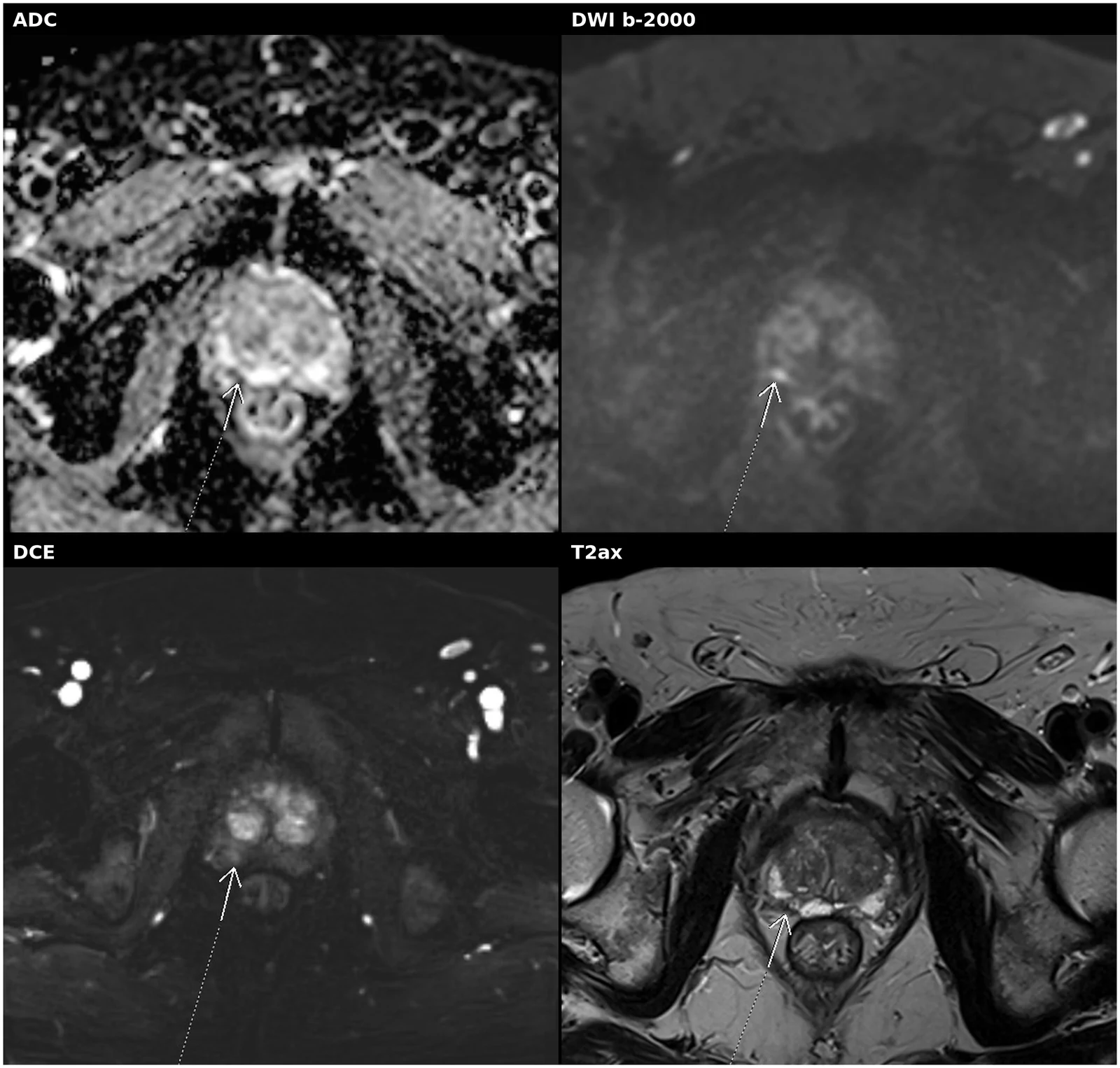
Multiparametric prostate MRI demonstrating a subtle focal lesion in the peripheral zone of the right lobe at the prostatic apex (arrow). The patient was 74 years old with a serum PSA level of 4.96 ng/mL and a PSAD of 0.072 ng/mL/cm³. The lesion exhibited mild-to-moderate diffusion restriction. Both fusion-guided biopsy and final histopathological examination following RARP confirmed a Gleason score of 3 + 3 (Grade Group 1). The mean ADC value was 850 × 10^−6^ mm²/s (range: 765–1068 × 10^−6^ mm²/s). MRI sequences: ADC map, DWI (b = 2000 s/mm²), DCE, T2ax.

**Table 1:** Distribution of ISUP grade groups (n = 192).

ISUP grade group	Gleason score	n	%
Grade Group 1	3 + 3	13	6.8
Grade Group 2	3 + 4	81	42.2
Grade Group 3	4 + 3	63	32.8
Grade Group 4	4 + 4, 3 + 5, 5 + 3	17	8.9
Grade Group 5	4 + 5, 5 + 4	18	9.4
Total	—	192	100.0

**Table 2:** Baseline clinical characteristics by ISUP grade group.

ISUP grade group	n	Age, years (mean ± SD)	PSA, ng/mL (median, IQR)	PSAD, ng/mL/mL (median, IQR)	Minimum ADC mean ± SD
1	13	63.5 ± 8.1	9.40 (5.29–13.51)	0.165 (0.107–0.260)	879 ± 135
2	81	66.9 ± 6.8	6.73 (5.27–9.20)	0.185 (0.125–0.269)	574 ± 152
3	63	68.3 ± 6.2	7.10 (5.63–9.36)	0.182 (0.127–0.284)	520 ± 139
4	17	71.2 ± 5.9	6.00 (4.82–10.00)	0.179 (0.102–0.290)	461 ± 140
5	18	66.3 ± 5.3	7.40 (6.51–9.40)	0.192 (0.121–0.340)	476 ± 150

### Ethics statement

This retrospective study analyzed anonymized clinical, imaging, and histopathological data obtained during routine diagnostic and therapeutic procedures. No interventions were performed for research purposes, and no identifiable patient information was accessed by the investigators. In accordance with institutional policies of the Medical University of Gdańsk regarding retrospective analyses of anonymized data, ethical review and approval were not required for this study. The study was conducted in accordance with the ethical principles outlined in the Declaration of Helsinki.

### Inclusion and exclusion criteria

Patients were eligible for inclusion if they underwent mpMRI on a 3.0-T scanner (Siemens Magnetom Vida, Siemens Healthineers, Germany), followed by systematic and/or targeted biopsy and subsequent robot-assisted radical prostatectomy (RARP), and had complete clinical, radiologic, and histopathologic data available. Patients were excluded if they had incomplete MRI datasets, underwent imaging on a 1.5-T scanner, or had significant DWI/ADC artifacts (e.g., due to hip prostheses or motion), or if they were not eligible for surgery or had incomplete histopathologic follow-up.

### Imaging protocol

Prostate mpMRI was performed following PI-RADS v2.1 recommendations and included axial, sagittal, and coronal high-resolution T2-weighted imaging; diffusion-weighted imaging with b-values of 0, 50, 1000, and 2000 s/mm², with ADC maps derived from b = 50, 1000, and 2000 s/mm²; and dynamic contrast-enhanced T1-weighted imaging acquired after administration of a gadolinium-based contrast agent (0.1 mmol/kg, injection rate 2 mL/s).

For ADC measurements, the lesion with the highest PI-RADS score was selected for quantitative analysis. Regions of interest (ROIs) were manually placed on the ADC map within the solid component of the lesion, avoiding areas of necrosis, hemorrhage, cystic degeneration, and visible artifacts. The ROI was positioned to encompass the largest representative portion of the lesion while remaining within its boundaries. The minimum ADC value was recorded as the lowest ADC value obtained within the lesion. MR examinations were evaluated by two experienced readers. ROI placement and ADC measurements were determined jointly during the evaluation of the examinations, and a single final ADC measurement, agreed upon by consensus, was recorded for each lesion.

### Statistical analysis

Statistical analyses were performed using SPSS v.29 (IBM Corp., Armonk, NY, USA) for descriptive statistics, the Kruskal–Wallis test, and Dunn–Bonferroni *post-hoc* comparisons across ISUP Grade Groups. Normality was assessed using the Shapiro–Wilk test; because minimum ADC, PSA, and PSA density deviated from a normal distribution, non-parametric methods were used throughout. Spearman’s rank correlation coefficient (ρ), with bootstrap-derived 95% confidence intervals, was used to evaluate monotonic relationships between minimum ADC, PSA, PSA density, age, and pathological ISUP Grade Group. Multivariable ordinal logistic regression was used to determine whether minimum ADC remained independently associated with ISUP Grade Group after adjustment for age, PSA (or PSA density), and prostate volume; because PSA density is mathematically derived from PSA and prostate volume, two separate models were constructed to avoid multicollinearity, since the variance inflation factors for PSA and PSA density in a combined model were 6.28 and 7.11, respectively. Receiver operating characteristic (ROC) curve analysis was used to evaluate the ability of minimum ADC, PSA, and PSA density, individually and in combination, to discriminate ISUP Grade Group 1 from ISUP Grade Group ≥2; areas under the curve (AUC) and their 95% confidence intervals were estimated using bootstrap resampling, and pairwise comparisons between models were performed using DeLong’s test. Because of the marked imbalance between outcome classes (13 vs. 179 patients), Precision–Recall curves and the corresponding area under the Precision–Recall curve (AUPRC) were additionally computed. A two-sided p value < 0.05 was considered statistically significant throughout.

### Biopsy-to-final pathology concordance analysis

To characterize the accuracy of pre-operative biopsy grading relative to final surgical pathology, each patient’s biopsy Gleason score was compared with the final Gleason score obtained at radical prostatectomy. Concordance was classified into three categories: concordant (biopsy and final Gleason score corresponding to the same ISUP Grade Group), upstaged (final ISUP Grade Group higher than the biopsy-derived Grade Group), and downstaged (final ISUP Grade Group lower than the biopsy-derived Grade Group). This classification was applied to the full 192-patient cohort and summarized both overall and stratified by final pathological ISUP Grade Group.

The individual biopsy strategy (systematic, fusion-guided/targeted, or combined) was not recorded at the patient level and could therefore not be used to stratify the concordance analysis; this is addressed as a limitation in the Discussion.

## Results

### Study population and baseline characteristics

A total of 192 patients were included in the study cohort. Pathological examination classified 13 patients as ISUP Grade Group 1, 81 as Grade Group 2, 63 as Grade Group 3, 17 as Grade Group 4, and 18 as Grade Group 5. Thus, 179 patients (93.2%) had ISUP Grade Group ≥2, whereas 13 patients (6.8%) had ISUP Grade Group 1.

The mean age of the study population was 67.5 ± 6.7 years. The median prostate volume was 38.2 mL (IQR, 29.6–51.0 mL). The median PSA concentration was 7.00 ng/mL (IQR, 5.30–9.66 ng/mL), and the median PSA density was 0.179 ng/mL/mL (IQR, 0.120–0.281 ng/mL/mL). Minimum ADC demonstrated a mean value of 558 ± 173 × 10^−6^ mm²/s.

The distribution of demographic, clinical, and imaging variables according to pathological ISUP Grade Group is presented in **Table 2**.

### Comparison of clinical and imaging parameters across ISUP Grade Groups

Minimum ADC values differed significantly across the five ISUP Grade Groups (Kruskal–Wallis H = 38.354, p < 0.001), with a progressive decrease in ADC as pathological grade increased. The mean minimum ADC was 879 ± 135 × 10^−6^ mm²/s in patients with ISUP Grade Group 1, compared with 574 ± 152 × 10^−6^ mm²/s in Grade Group 2, 520 ± 139 × 10^−6^ mm²/s in Grade Group 3, 461 ± 140 × 10^−6^ mm²/s in Grade Group 4, and 476 ± 150 × 10^−6^ mm²/s in Grade Group 5.

Mean ADC values also differed significantly across ISUP Grade Groups (H = 40.907, p < 0.001), with progressively lower values observed in higher-grade disease.

In contrast, PSA did not differ significantly across ISUP Grade Groups (H = 2.837, p = 0.585). Similarly, PSA density showed no significant difference between the pathological Grade Groups (H = 0.694, p = 0.952). Prostate volume also did not differ significantly across the five groups (H = 7.402, p = 0.116).

Age differed significantly between ISUP Grade Groups (H = 11.405, p = 0.022). However, subsequent correlation analyses (below) did not demonstrate a significant association between age and either PSA or PSA density, arguing against age as a confounder of the PSA–grade relationship.

Detailed descriptive statistics and subgroup sizes are presented in **Table 2**.

### Correlation analysis

Spearman rank correlation analysis was performed to evaluate the monotonic relationships between minimum ADC, PSA, PSA density, age, and pathological ISUP Grade Group.

Minimum ADC demonstrated a statistically significant inverse correlation with ISUP Grade Group (Spearman’s ρ = −0.355; 95% CI, −0.473 to −0.218; p < 0.001), indicating that lower ADC values were associated with higher pathological tumor grade.

No significant monotonic relationship was observed between PSA and ISUP Grade Group (ρ = 0.012; 95% CI, −0.134 to 0.161; p = 0.864). Similarly, PSA density was not significantly correlated with ISUP Grade Group (ρ = 0.040; 95% CI, −0.109 to 0.185; p = 0.578).

To assess whether age could represent a potential confounding factor in the PSA-based analyses, correlations between age and PSA, and between age and PSA density, were also evaluated. Neither association was statistically significant: age versus PSA, ρ = −0.040 (95% CI, −0.180 to 0.103; p = 0.582); age versus PSA density, ρ = −0.055 (95% CI, −0.199 to 0.086; p = 0.443). Thus, the lack of association between PSA-based parameters and pathological grade in this cohort does not appear to be explained by the correlation of age with PSA or PSA density.

The complete correlation analysis is presented in **Table 3**.

**Table 3:** Spearman rank correlation analysis (n = 192).

Variable pair	Spearman’s ρ	95% CI	p
Minimum ADC – ISUP Grade Group	−0.355	−0.473 to −0.218	<0.001
PSA – ISUP Grade Group	0.012	−0.134 to 0.161	0.864
PSA density – ISUP Grade Group	0.040	−0.109 to 0.185	0.578
Age – PSA	−0.040	−0.180 to 0.103	0.582
Age – PSA density	−0.055	−0.199 to 0.086	0.443

### Multivariable ordinal logistic regression

Multivariable ordinal logistic regression was performed to determine whether minimum ADC remained independently associated with pathological ISUP Grade Group after adjustment for relevant clinical variables.

Because PSA density is mathematically derived from PSA and prostate volume, simultaneous inclusion of PSA, PSA density, and prostate volume in the same model would introduce substantial multicollinearity. Therefore, two complementary multivariable models were evaluated.

Model 1 included minimum ADC, age, PSA, and prostate volume. Minimum ADC remained independently associated with higher ISUP Grade Group (OR = 0.995 per 1 × 10^−6^ mm²/s increase; 95% CI, 0.993–0.996; p < 0.001). Age was not independently associated with ISUP Grade Group (OR = 1.039; 95% CI, 0.998–1.082; p = 0.064), nor were PSA (OR = 1.017; 95% CI, 0.976–1.060; p = 0.425) or prostate volume (OR = 0.995; 95% CI, 0.982–1.009; p = 0.492).

Model 2 included minimum ADC, age, PSA density, and prostate volume. Minimum ADC again remained independently associated with higher ISUP Grade Group (OR = 0.995 per 1 × 10^−6^ mm²/s increase; 95% CI, 0.993–0.996; p < 0.001). Age (OR = 1.038; 95% CI, 0.997–1.081; p = 0.071), PSA density (OR = 0.997; 95% CI, 0.989–1.005; p = 0.506), and prostate volume (OR = 0.996; 95% CI, 0.983–1.009; p = 0.553) were not statistically significant.

Thus, the inverse association between minimum ADC and pathological tumor grade remained significant after adjustment for age, PSA or PSA density, and prostate volume, in both models.

The results of the multivariable models are summarized in **Table 4**.

**Table 4:** Multivariable ordinal logistic regression models for ISUP grade group (n = 192).

Model	Variable	OR	95% CI	p
Model 1: ADC + age + PSA + prostate volume	Minimum ADC	0.995	0.993–0.996	<0.001
	Age	1.039	0.998–1.082	0.064
	PSA	1.017	0.976–1.060	0.425
	Prostate volume	0.995	0.982–1.009	0.492
Model 2: ADC + age + PSA density + prostate volume	Minimum ADC	0.995	0.993–0.996	<0.001
	Age	1.038	0.997–1.081	0.071
	PSA density	0.997	0.989–1.005	0.506
	Prostate volume	0.996	0.983–1.009	0.553

### ROC analysis

Receiver operating characteristic (ROC) analysis was performed to evaluate the ability of minimum ADC, PSA, and PSA density to discriminate between ISUP Grade Group 1 and ISUP Grade Group ≥2.

Minimum ADC demonstrated high discriminatory performance, with an AUC of 0.959 (95% CI, 0.923–0.988). In comparison, PSA demonstrated an AUC of 0.586 (95% CI, 0.376–0.785), while PSA density demonstrated an AUC of 0.562 (95% CI, 0.392–0.729).

The individual-marker ROC curves are presented in **Figure 5**. The corresponding AUC values and 95% confidence intervals (bootstrap-based) are reported in **Table 5**.

**Figure 5:**
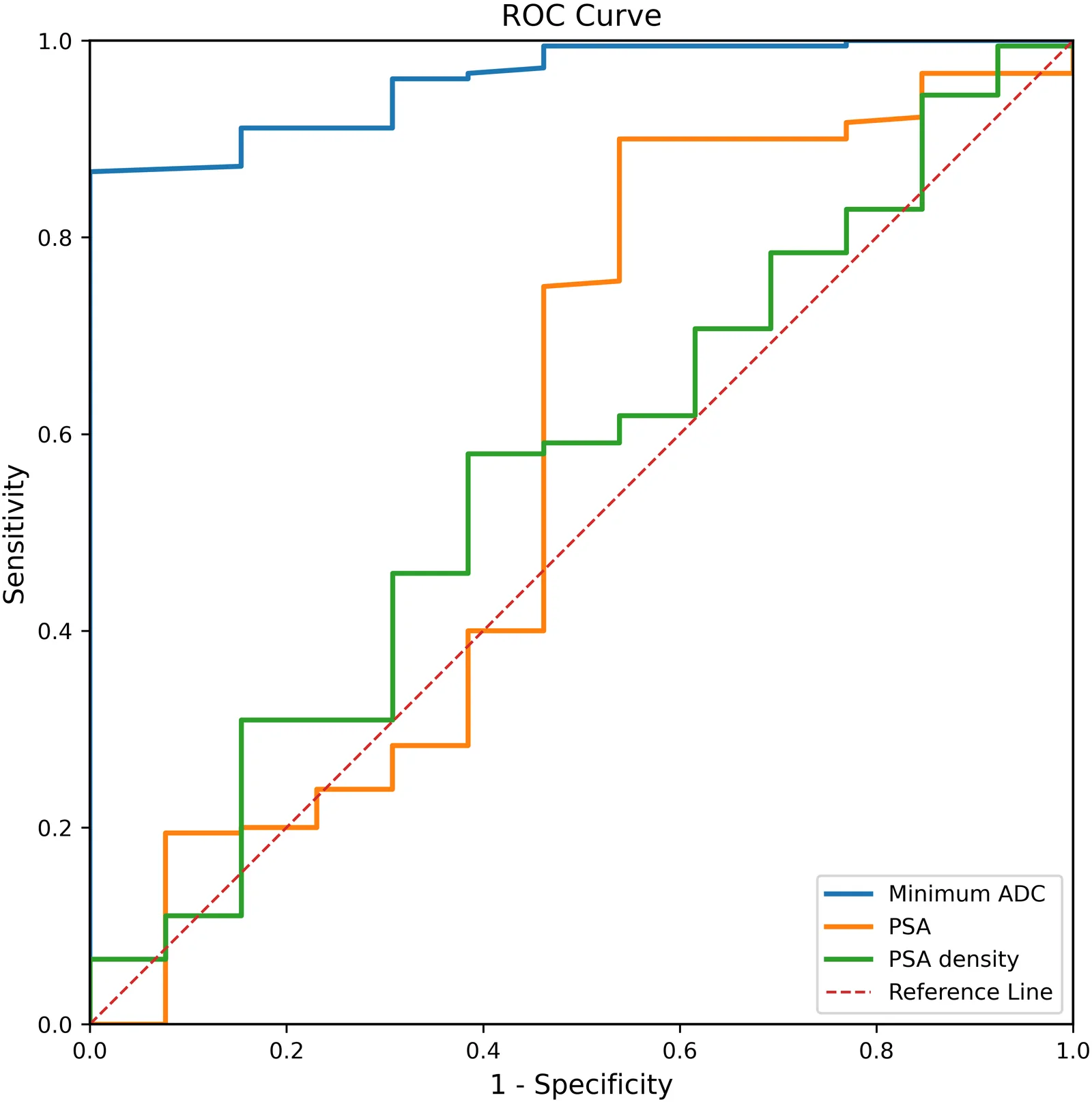
Receiver operating characteristic (ROC) curves for discrimination between ISUP Grade Group 1 and ISUP Grade Group ≥2 for the individual markers minimum ADC, PSA, and PSA density. AUC values and 95% confidence intervals are reported in **Table 5**.

**Table 5:** Diagnostic performance (ROC analysis) for discrimination between ISUP grade group 1 and ISUP grade group ≥2 (n = 192).

Marker/model	AUC	95% CI
Minimum ADC	0.959	0.921–0.988
PSA	0.586	0.339–0.785
PSA density	0.562	0.386–0.723
ADC + PSA	0.961	0.922–0.989
ADC + PSA density	0.959	0.921–0.988
ADC + PSA + PSA density	0.977	0.927–0.99

95% CIs were derived using bootstrap resampling.

### Combined prediction models

To determine whether PSA-based variables provided incremental discriminatory information beyond minimum ADC, combined logistic regression models were evaluated.

The combination of minimum ADC and PSA demonstrated an AUC of 0.961 (95% CI, 0.924–0.990). The combination of minimum ADC and PSA density demonstrated an AUC of 0.959 (95% CI, 0.924–0.987). The model incorporating minimum ADC, PSA, and PSA density demonstrated the highest numerical AUC, at 0.963 (95% CI, 0.927–0.990).

Although the three-variable model demonstrated a numerically higher AUC than minimum ADC alone, formal comparison using DeLong’s test did not demonstrate a statistically significant improvement in discrimination (ADC vs. ADC + PSA + PSA density: p = 0.102). Similarly, adding PSA alone (ADC vs. ADC + PSA: p = 0.626) or PSA density alone (ADC vs. ADC + PSA density: p = 0.727) to minimum ADC did not result in a statistically significant improvement in AUC. In contrast, adding ADC to PSA (PSA vs. ADC + PSA: p < 0.001) or to PSA density (PSA density vs. ADC + PSA density: p < 0.001) produced a highly significant improvement, confirming that ADC — rather than the PSA-based parameters — drives the discriminatory performance of the combined models.

The ROC curves for the combined models are shown in **Figure 6**, and the corresponding AUC values and 95% confidence intervals are summarized in **Table 5**.

**Figure 6:**
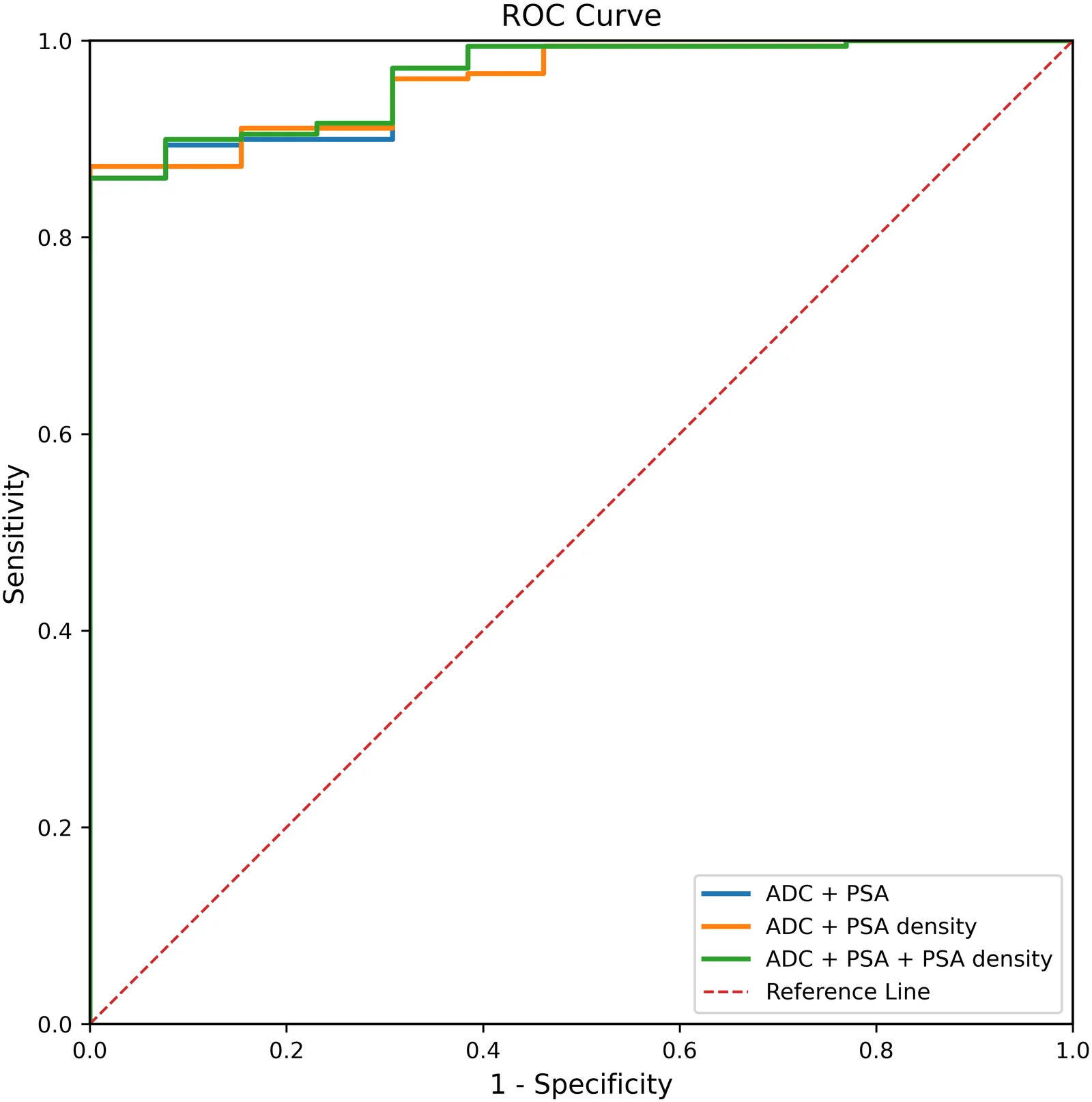
Receiver operating characteristic (ROC) curves for combined models for discrimination between ISUP Grade Group 1 and ISUP Grade Group ≥2. Models included ADC + PSA, ADC + PSA density, and ADC + PSA + PSA density. AUC values and 95% confidence intervals are reported in **Table 5**.

### Precision–recall analysis

Because of the marked imbalance between the two outcome classes, Precision–Recall analysis was additionally performed.

In our analysis, ISUP Grade Group ≥2 was defined as the positive class, while ISUP Grade Group 1 was considered the negative class. The positive class represented 93.2% of the study cohort; therefore, the no-skill baseline AUPRC was 0.932.

Minimum ADC demonstrated a high area under the Precision–Recall curve (AUPRC = 0.997), supporting its strong discriminative performance in distinguishing ISUP Grade Group 1 from ISUP Grade Group ≥2. PSA (AUPRC = 0.935) and PSA density (AUPRC = 0.946) demonstrated lower Precision–Recall performance compared with minimum ADC. The combined models showed AUPRC values of 0.997 (ADC + PSA), 0.997 (ADC + PSA density), and 0.998 (ADC + PSA + PSA density). **Table 6**.

**Table 6:** Area under the precision–recall curve (AUPRC) for minimum ADC, PSA, PSA density, and their combined models in distinguishing ISUP grade group 1 from ISUP grade group ≥2.

Model	AUPRC
ADC	0,997
PSA	0,935
PSA density	0,946
ADC + PSA	0,997
ADC + PSA density	0,997
ADC + PSA + PSA density	0,998

The Precision–Recall results were interpreted in the context of the outcome distribution, in which only 13 patients had ISUP Grade Group 1 compared with 179 patients with ISUP Grade Group ≥2. Therefore, the high AUPRC values should be interpreted cautiously, as the prevalence of the positive class substantially influences the Precision–Recall curve.

### Overall findings

Overall, minimum ADC demonstrated a consistent and statistically significant inverse relationship with pathological ISUP Grade Group. Lower ADC values were associated with higher pathological tumor grade, and this association remained significant after adjustment for age, PSA or PSA density, and prostate volume.

Neither PSA nor PSA density demonstrated a significant monotonic correlation with ISUP Grade Group. Furthermore, age was not significantly correlated with either PSA or PSA density, providing no evidence that age accounted for the lack of association between PSA-based parameters and pathological grade.

Minimum ADC also demonstrated substantially greater discriminatory performance than PSA or PSA density in ROC analysis. Although combined models incorporating ADC and PSA-based parameters yielded numerically similar or slightly higher AUC values, no statistically significant improvement over minimum ADC alone was demonstrated.

Given the small number of patients with ISUP Grade Group 1, these diagnostic performance estimates should be interpreted cautiously and require confirmation in larger, independently validated cohorts.

### Biopsy-to-final pathology concordance

Among the 192 patients in the cohort, biopsy and final pathological grading were concordant in 79 patients (41.1%), while 75 patients (39.1%) were upstaged and 38 patients (19.8%) were downstaged at final pathology relative to the biopsy Gleason score.

Concordance varied by final ISUP Grade Group (**Figure 7**). No patient was upstaged into ISUP Grade Group 1 by definition (0 of 13); 8 of 13 (61.5%) were concordant and 5 of 13 (38.5%) were downstaged from a higher biopsy grade. In contrast, upstaging predominated among patients with final ISUP Grade Group 3 (36 of 63, 57.1%) and Grade Group 5 (13 of 18, 72.2%). Among patients with a biopsy Gleason score of 3 + 3 (n = 38), only 8 (21.1%) remained ISUP Grade Group 1 at final pathology, while 30 (78.9%) were upstaged, including 3 patients upstaged to ISUP Grade Group 4 or 5.

**Figure 7:**
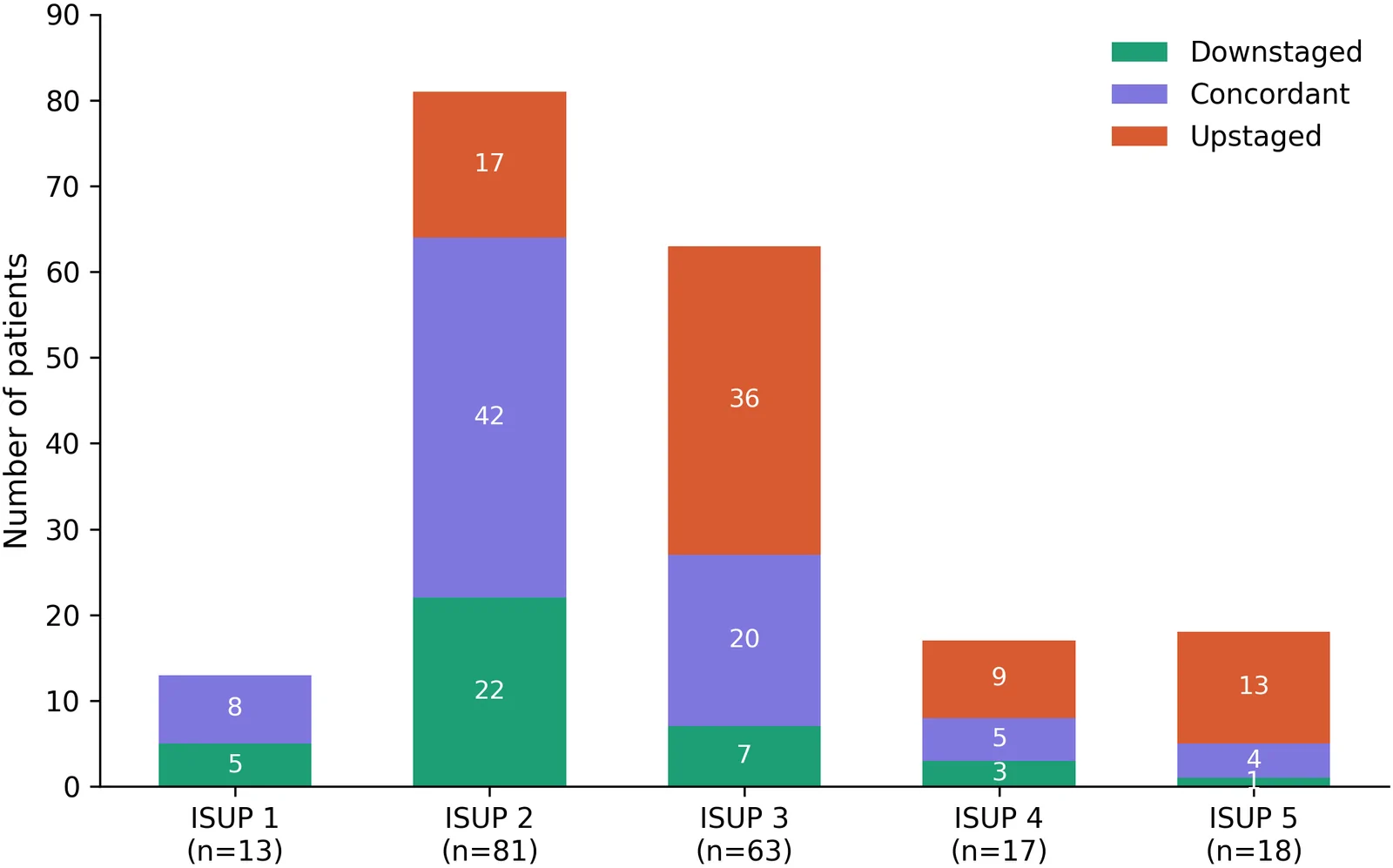
Concordance between biopsy Gleason score and final pathological ISUP grade group after radical prostatectomy (n = 192), stratified by final ISUP grade group. Bars show the number of patients whose biopsy grading was downstaged, concordant, or upstaged relative to final pathology within each final grade group.

These findings confirm substantial grade discordance between biopsy and final pathology in this surgical cohort, particularly pronounced undergrading of biopsy Gleason 3 + 3 disease, consistent with the rationale for evaluating minimum ADC as a complementary, biopsy-independent marker of tumor grade.

## Discussion

Our analysis demonstrated a robust inverse relationship between minimum ADC and pathological ISUP Grade Group, confirmed by correlation analysis, multivariable ordinal logistic regression, and ROC analysis, while serum PSA and PSA density failed to show a consistent association with grade in this cohort. This finding underscores the growing role of quantitative MRI biomarkers in the functional assessment of prostate cancer, complementing and, in this cohort, outperforming biochemical indices in reflecting tumor aggressiveness and histoarchitectural disruption. The inverse correlation between ADC and Gleason grade has now been repeatedly confirmed across numerous studies, including high-field 3.0 T MRI analyses and meta-analyses that integrate thousands of prostate cancer lesions (8, 13–15, 17). Our data are consistent with these reports, supporting the concept that ADC not only reflects tissue cellularity but also captures the complex microstructural reorganization that accompanies dedifferentiation and loss of glandular architecture in high-grade tumors.

Histologically, a progressive reduction in glandular lumen and stromal rearrangement contributes more strongly to ADC decline than cellular density alone, suggesting that diffusion-weighted imaging probes tumor tissue organization at a microscopic level (18, 19). High-grade adenocarcinomas, characterized by fused glands and solid sheets of malignant cells, exhibit marked restriction of water diffusivity, resulting in significantly lower ADC values. From a biological standpoint, ADC can be viewed as an imaging surrogate for the histologic “disappearance of glandular identity,” a key determinant of Gleason pattern progression. Voxel-based and histogram analyses have further refined this understanding: the lower 10th percentile ADC within a lesion, representing the most densely packed tumor components, correlates more strongly with ISUP grade groups than mean ADC (18). This illustrates that spatial heterogeneity within prostate cancer lesions can be quantitatively captured, allowing radiologists to infer microscale features that previously required histopathology for confirmation.

In contrast to ADC, PSA and PSA density showed poor discriminatory ability across ISUP Grade Groups in our cohort, with essentially no monotonic correlation with pathological grade. Although PSA has been instrumental in prostate cancer screening for decades (3, 5), it mainly reflects glandular epithelial secretion rather than true tumor aggressiveness (6, 20). Many high-grade, poorly differentiated tumors demonstrate markedly reduced PSA production despite substantial tumor volumes (6). This paradox highlights the limitation of conventional PSA-based risk assessment. Notably, in our combined models, adding ADC to PSA or PSA density produced a highly significant improvement in discrimination, whereas adding PSA or PSA density to ADC did not — indicating that, in this cohort, ADC captured essentially all of the discriminatory information available, with PSA-based parameters contributing little incremental value.

The present findings have direct, though still preliminary, clinical relevance. Biopsy-to-final pathology concordance in this cohort was limited: only 41.1% of patients had concordant grading, and among patients with a biopsy Gleason score of 3 + 3, 78.9% were upstaged at final pathology (**Figure 7**). Because minimum ADC was independently and inversely associated with final pathological grade, it is a plausible candidate marker for flagging which biopsy-3 + 3 patients are most likely to harbor occult higher-grade disease — for example, a lesion with an unexpectedly low ADC despite a low-grade biopsy result. However, we did not formally test whether ADC discriminates upstaged from concordant patients within the biopsy-3 + 3 subgroup, since this requires linking individual ADC values to biopsy-versus-final concordance status, which was beyond the scope of the present analysis; this is an important direction for follow-up work using the same cohort. This cross-sectional, radical-prostatectomy cohort also did not evaluate patients longitudinally during active surveillance. These specific applications, discussed further below with reference to the existing literature, should therefore be regarded as hypothesis-generating rather than as findings established by the present data.

Other groups have examined the role of ADC specifically in patients undergoing active surveillance, a population and study design distinct from ours. Henderson et al. observed that men with lower baseline ADC required earlier radical treatment and had shorter time to adverse histology in a prospective active-surveillance cohort (21). Likewise, longitudinal studies have suggested that serial ADC decline of ≥10% combined with lesion volume increase of ≥20% may serve as an objective trigger for re-biopsy, and standardized frameworks such as the PRECISE scoring system have been proposed to bring quantitative precision to MRI-based surveillance reporting (22–24).

We cite this literature for context; our own cross-sectional, surgically selected cohort did not evaluate longitudinal ADC change or surveillance outcomes, and these findings should not be extrapolated directly from the present data.

We deliberately do not propose a specific numeric ADC cut-off for distinguishing ISUP Grade Group 1 from ≥2 in this manuscript. Given the small number of ISUP Grade Group 1 patients (n = 13), any Youden-index-derived threshold would carry a high risk of instability and overinterpretation, and previously published ADC thresholds vary widely depending on the ADC metric (mean vs. minimum/lowest-percentile ADC), b-value combinations, field strength, scanner vendor, and ROI methodology (13, 15, 17, 18), limiting the value of a single numeric comparison. We consider the consistent direction and strength of the inverse ADC–grade relationship demonstrated here, rather than a specific cut-off value, to be the primary finding of this study; establishing a clinically applicable threshold will require dedicated, adequately powered, prospective cut-off-validation studies, ideally using a harmonized acquisition protocol across centers.

Despite these advantages, several limitations must be noted. Our analysis was retrospective and single-center in design, and although imaging parameters followed PI-RADS v2.1 recommendations (11), inter-scanner and inter-observer variability in ADC measurements remain potential confounders. In addition, the cohort was restricted to patients who ultimately underwent radical prostatectomy, representing a surgically selected population; this selection may limit generalizability to the broader population of men undergoing biopsy or being considered for active surveillance, and it should be taken into account when interpreting the diagnostic performance estimates reported here.

A further limitation concerns biopsy technique. Patients underwent systematic and/or MRI/ultrasound fusion-guided (targeted) biopsy per the inclusion criteria, but the specific biopsy strategy used in each individual patient was not recorded at the patient level and could therefore not be used to stratify the biopsy-to-final pathology concordance analysis reported above. Combined systematic-plus-targeted biopsy has been shown to reduce grade discordance relative to systematic biopsy alone (25); consequently, the upstaging and downstaging rates reported here reflect a mixed, real-world biopsy strategy consistent with routine clinical practice at our center, rather than the performance of a single, uniform biopsy technique, and should not be generalized to cohorts relying exclusively on systematic or exclusively on targeted biopsy. Prospective studies that record biopsy technique at the patient level will be needed to determine whether ADC’s association with grade, and its potential value for flagging under-sampled disease, differs by biopsy approach.

Standardization of diffusion sequences, b-values, and region-of-interest placement across institutions remains an unmet prerequisite for widespread adoption of ADC thresholds in clinical decision-making. The reproducibility of ADC measurements is generally high in the peripheral zone but weaker in the transition zone, where benign prostatic hyperplasia increases background heterogeneity (25, 26). The number of ISUP Grade Group 1 patients in our cohort was small (n = 13), which limits the precision of the diagnostic performance estimates for this subgroup despite the use of bootstrap resampling for confidence intervals; this should be regarded as an important limitation requiring confirmation in larger cohorts. Another limitation of this study is the lack of formal lesion-level correlation between MRI findings and histopathology. Although radiologists had access to the histopathological report following radical prostatectomy, no direct correlation with microscopic (histological) images was performed, and no formal matching was carried out between the MRI-detected lesion selected for ADC measurement and the corresponding tumor focus in the surgical specimen. Consequently, the possibility of discordance between the MRI-defined lesion and the histopathologically confirmed tumor focus cannot be excluded, which may have introduced a degree of uncertainty into the reported ADC values. Future studies incorporating systematic radiological-pathological correlation, such as whole-mount step-sectioning with image co-registration, would allow for more precise lesion-level validation.

To mitigate these limitations, future multicenter prospective studies employing harmonized scanning protocols, formal biopsy-to-prostatectomy upstaging analysis, and voxel-wise analysis are essential. Additionally, integration of ADC with other quantitative MRI parameters such as T2 texture features, diffusion kurtosis, or radiomics signatures may further improve risk stratification accuracy (8, 27, 28, 29).

In conclusion, in this radical prostatectomy cohort, minimum ADC was inversely and independently associated with pathological ISUP Grade Group, whereas serum PSA and PSA density were not. The present data demonstrate a strong association between ADC and pathological tumor grade in a selected surgical population, but they do not establish ADC as an independent clinical tool for active-surveillance management, biopsy-upstaging prediction, or longitudinal disease monitoring, as these applications were not directly evaluated in this cross-sectional study. ADC should therefore be regarded as a promising, complementary quantitative biomarker rather than a stand-alone decision-making tool. Prospective, multicenter studies incorporating standardized acquisition protocols, biopsy-to-prostatectomy upstaging analysis, and longitudinal follow-up are needed before ADC-based thresholds, alone or integrated with PSA density, lesion volume, PI-RADS score, or radiomic features, can be recommended for routine risk stratification in localized prostate cancer.

## Data Availability

The raw data supporting the conclusions of this article will be made available by the authors, without undue reservation.
